# Circulating ghrelin levels in patients with gastric cancer: a systematic review and meta-analysis

**DOI:** 10.3389/fonc.2023.1255112

**Published:** 2023-09-15

**Authors:** Yuxuan Wang, Caishun Zhang, Jiaqing Yu, Qing Zhang, Yukai Wang, Yunqiu Xia, Jing Dong

**Affiliations:** ^1^ Clinical Medicine Department, College of Basic Medicine, Qingdao University, Qingdao, China; ^2^ College of Nursing, Qingdao University, Qingdao, China; ^3^ Special Medicine Department, College of Basic Medicine, Qingdao University, Qingdao, China; ^4^ Laboratory of Human Body Function, School of Basic Medicine, Qingdao University, Qingdao, China; ^5^ Physiology Department, College of Basic Medicine, Qingdao University, Qingdao, China

**Keywords:** meta-analysis, ghrelin, gastric cancer, oncology, hunger hormone

## Abstract

**Background:**

Ghrelin plays a critical role in regulating energy metabolism and homeostasis. The association between circulating ghrelin levels and gastric cancer has not been systematically analyzed.

**Objective:**

This work explored the association between circulating ghrelin levels and gastric cancer.

**Methods:**

The literature search for relevant articles published until November 2022 was performed using PubMed, Cochrane Library, EMBASE, and Web of Science with the keywords “ghrelin” and “gastric cancer”. Standardized mean differences (SMD) with 95% confidence intervals were used to measure the effectiveness. We assessed pooled data by use of a random-effects model.

**Results:**

Of 5,302 identified studies, nine were included (N=3,196 participants). Circulating ghrelin levels were lower in gastric cancer patients (SMD=-0.255, 95%CI: -0.528 to 0.017, P < 0.00001), but with high heterogeneity (I^2 ^= 88.8%).

**Conclusion:**

The circulating ghrelin levels in patients with gastric cancer were lower than in controls. However, there was heterogeneity among results; therefore, studies with larger sample sizes are recommended.

## Introduction

GLOBOCAN 2020 estimated 9,958,133 cancer deaths and 19,292,789 cancer cases globally (1). According to statistics, gastric cancer was the fifth most common cancer, with 1.09 million new cases (5.6%), and the fourth most common cause of cancer death, with 0.76 million deaths (7.7%) globally (1).

Gastric cancer is characterized by complex genetic and environmental interactions contributing to its initiation and progression (2). Risk factors included *Helicobacter pylori* infection, advanced age, male, smoking, high salt intake, and diets low in fruit and vegetables (3–5). The most common cause of sporadic distal gastric cancer is *H. pylori* infection (3, 4). In addition, many gastrointestinal hormones are associated with gastric cancer (6–8).

Ghrelin is an endogenous orexigenic peptide hormone of 28 amino acids that binds to the growth hormone secretagogue receptor 1α. Kojima et al. first reported this hormone (9–11). Ghrelin is secreted by the oxyntic glands of the stomach and GHSR is expressed in pituitary gland, hypothalamus, lung, kidney, liver, adipose tissue and endocrine pancreas (9, 12). The acylation of ghrelin is essential for binding and activating its receptor but most (80–90%) circulating ghrelin is non-acylated (12, 13). Ghrelin performs several physiological functions, including orexigenic effect, growth hormone secretion stimulation, insulin secretion inhibition, and anti-inflammatory activity (9, 12, 14–16). Reviews of the role of ghrelin in cancers established associations between ghrelin and tumor progression in many different tumor types (17–20). Other studies found that circulating ghrelin levels in patients with gastric cancer decreased after gastrectomy (21, 22). These findings suggest a strong link between ghrelin and gastric cancer.

We noticed that some clinical studies found patients with gastric cancer have low circulating ghrelin levels (21–23); however, other studies reached the opposite conclusions (24, 25). Therefore, the aim of our study was to explore the association between circulating ghrelin levels and gastric cancer.

## Materials and methods

### Date source and search strategy

We selected relevant studies published to November 1, 2022 by searching PubMed, Cochrane Library, EMBASE and Web of Science. Medical subject headings included “ghrelin” and “tumor”. We searched the following free terms in pubMed: Neoplasias, Neoplasm, Tumors, Cancer, Malignancy, Neoplasia, Malignancy, Malignant Neoplasms, Cancers, Malignant Neoplasm, Benign Neoplasm, GHRL Protein, Benign Neoplasms, Ghrelin-Obestatin Preprohormone, Ppghrelin, Ghrelin Obestatin Preprohormone, Motilin Related Peptide Precursor, Peptide Precursor, Motilin-Related, Precursor, Motilin-Related Peptide Precursor, Motilin-Related Peptide, Ghrelin Precursor, PpMTLRP, Precursor, Obestatin, Appetite-Regulating Hormone, Ghrelin, Motilin-Related Peptide, Motilin Related Peptide Appetite Regulating Hormone, and Gastric MLTRP.

### Study selection and criteria

Eligible studies met the following criteria: (1) subjects were all adults; (2) studied gastric cancer; (3) subjects included both gastric cancer patients and controls; (4) original articles with≥20 subjects; (5) published in English.

The excluded research met the following criteria: (1) studies on animals; (2) studies without controls; (3) studies with substantial statistical errors or unreliable designs; (4) meta-analysis, reviews, comments and letters.

### Data extraction and quality assessment

All studies were reviewed by 2 independent reviewers (Wang YX and Zhang CS) and data were extracted in a standardized format. The extracted data were as follows: study information (author, country, published year, number of men, and women study population); and subject characteristics (ghrelin levels, BMI, age, ghrelin type). The ghrelin levels were converted to unified units (pg/mL) as needed.

In the case-control study, we assessed three items using the Newcastle-Ottawa Scale (NOS): A: whether the definitions of gastric cancer were adequate; B: whether the cases were representative; C: whether the control groups were from the same community; D: whether the control subjects had a history of disease; E: Whether the designs or analyses were comparable between cases and controls; F: whether ascertainment of exposure included secure records or structured interviews that were blind to case/control status; G: whether cases and controls were ascertained identically; and H: whether the cases and controls showed identical non-response rates. An asterisk is assigned to each parameter, 0 (lowest) to 8 (highest). Studies with a score ≥7 were considered high quality, and other studies were classified as moderate quality.

Our meta-analysis includes data presented as an abstract in a meeting (26).

### Data synthesis and analysis

Comparisons of ghrelin levels between patients with gastric cancer and controls were analyzed using a random effects model, which used mean values and standard deviations. We used standard mean difference and 95% confidence intervals to analyze continuous variables. Cochran’s (chi-square) test to measure heterogeneity and the I^2^ statistic to determine the extent of consistency: an I^2^ of over 75% indicates a high level of inconsistency, I^2^ of above 50% is moderate, and I^2^ of below 25% is low (27). Differences with p-values less than 0.05 were considered statistically significant. Subgroup analysis was performed according to the ghrelin type and race. Publication bias was assessed using Egger’s regression asymmetry test. Stata MP software (Version 17.0) was used for statistical analysis.

## Results

### The study inclusion procedure and study characteristics

**Figure 1:**
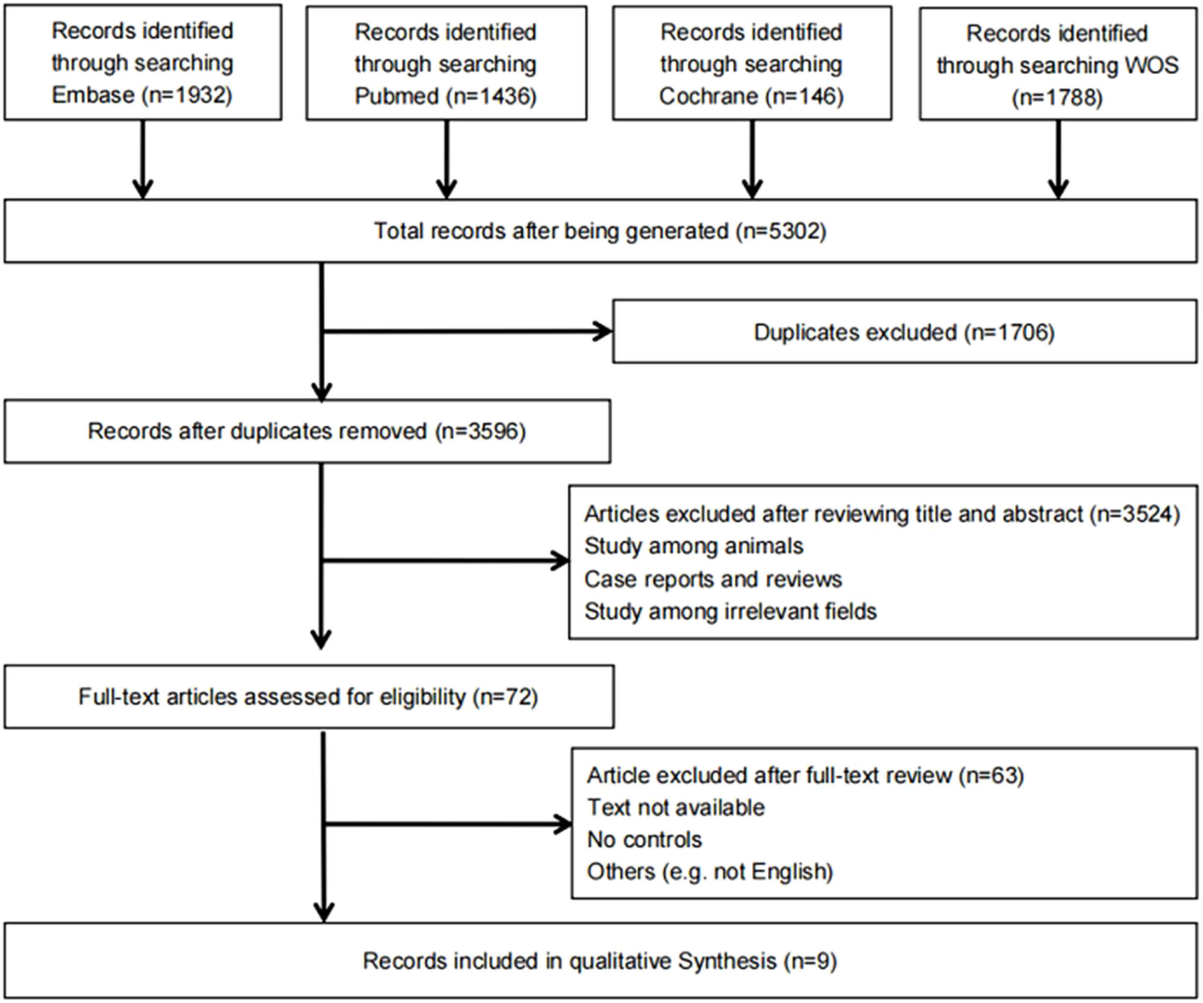
Flow chart of literature search.

The detailed steps of study screening are shown in **Figure 1**. We extracted 5,302 potential literatures from PubMed, Embase, Cochrane Library, and the Web of Science. After a duplication check, 1,706 studies were removed. After review of titles and abstracts, 3,524 ineligible studies were removed. After review of the full text, 63 studies were removed. A total of 9 studies were included (21–24, 26, 28–31).

**Tables 1**, **2** summarize the characteristics of the included studies. Pritchett et al. studied two groups in different regions in China (23). A total of 9 studies involving 10 groups and 3,196 patients were included. The studies were published from 2005 to 2022 and the sample sizes ranged from 27 to 1546. All subjects were adults. Each group was divided into those with gastric cancer and healthy controls. Three studies included subjects from Europe (21, 26, 28), and there were six from Asia (22–24, 29–31). Blood samples were taken under fasting conditions in six of these studies (21, 22, 28–31), and not stated in the remaining three studies (23, 24, 26). Blood samples were obtained from serum in one study (24) while the remaining eight were obtained from plasma. All the studies measured non-acylated ghrelin.

**Table 1:** Characteristics of included studies.

Author. Year	Race	Participants	Number	Blood sample	Gender (Male/Female)	Age	BMI	Ghrelin
Changzhen Zhu et al., 2020	Asian	Gastric cancer	38	Serum	29/9	Unspecified	Unspecified	22.4 ± 6.2
Qi Huang-Yue et al., 2007	Asian	Gastric cancer	58	Plasma	43/15	58.9 ± 13.3	21.8 ± 3.4	110.3 ± 92.1
Natalie R. Pritchett et al., 2020	Asian	Gastric cancer	776	Plasma	467/309	55.3 ± 8	21.7 ± 2.2	917 ± 355.6
Natalie R. Pritchett et al., 2020	Asian	Gastric cancer	249	Plasma	Unspecified	58 ± 9	24.6 ± 3.3	706 ± 259
Hai-Tao Wang et al., 2008	Asian	Gastric cancer	42	Plasma	19/23	49.5 ± 6	24.6 ± 3.3	464.6 ± 126.5
Anna Zub-Pokrowiecka et al., 2011	European	Gastric cancer	25	Plasma	14/11	49.5 ± 6	23.4 ± 2.8	191.1 ± 17.8
Krike P. et al., 2018	European	Gastric cancer	196	Plasma	Unspecified	Unspecified	Unspecified	464.2 ± 268
O Kemik et al., 2011	European	Gastric cancer	31	Plasma	14/17	46.8 ± 11.9	16.2 ± 1.5	723.7 ± 270.8
Hajime Isomoto et al., 2005	Asian	Gastric cancer	23	Plasma	11/12	60	22.1	183.2 ± 121.2
Hye-Kyung Jung et al., 2022	Asian	Gastric cancer	13	Plasma	8/5	59.2 ± 10.4	24.6 ± 2.8	631 ± 97

**Table 2:** Characteristics of included studies.

Author. Year	Race	Participants	Number	Blood sample	Gender (Male/Female)	Age	BMI	Ghrelin
Changzhen Zhu et al., 2020	Asian	Controls	69	Serum	Unspecified	Unspecified	Unspecified	19 ± 5.8
Qi Huang-Yue et al., 2007	Asian	Controls	24	Plasma	18/6	56.7 ± 12	24.1 ± 3.8	91.4 ± 73.6
Natalie R. Pritchett et al., 2020	Asian	Controls	770	Plasma	255/515	51 ± 9	22.1 ± 2.6	1022 ± 386
Natalie R. Pritchett et al., 2020	Asian	Controls	498	Plasma	Unspecified	58 ± 9	24.8 ± 3.6	743 ± 245
Hai-Tao Wang et al., 2008	Asian	Controls	20	Plasma	8/12	40.4 ± 10.2	22.2 ± 2.2	472 ± 115.9
Anna Zub-Pokrowiecka et al., 2011	European	Controls	25	Plasma	12/13	49.3 ± 11.5	23.8 ± 2.7	302.7 ± 76.7
Krike P. et al., 2018	European	Controls	246	Plasma	Unspecified	Unspecified	Unspecified	545.9 ± 275.1
O Kemik et al., 2011	European	Controls	40	Plasma	22/18	40.4 ± 11.3	21.5 ± 2.0	1104.3 ± 201.3
Hajime Isomoto et al., 2005	Asian	Controls	39	Plasma	16/23	51	22.6	174.8 ± 125.1
Hye-Kyung Jung et al., 2022	Asian	Controls	14	Plasma	7/7	49.6 ± 5.8	22.5 ± 2.3	555.8 ± 98.4

### Overall analysis

**Figure 2:**
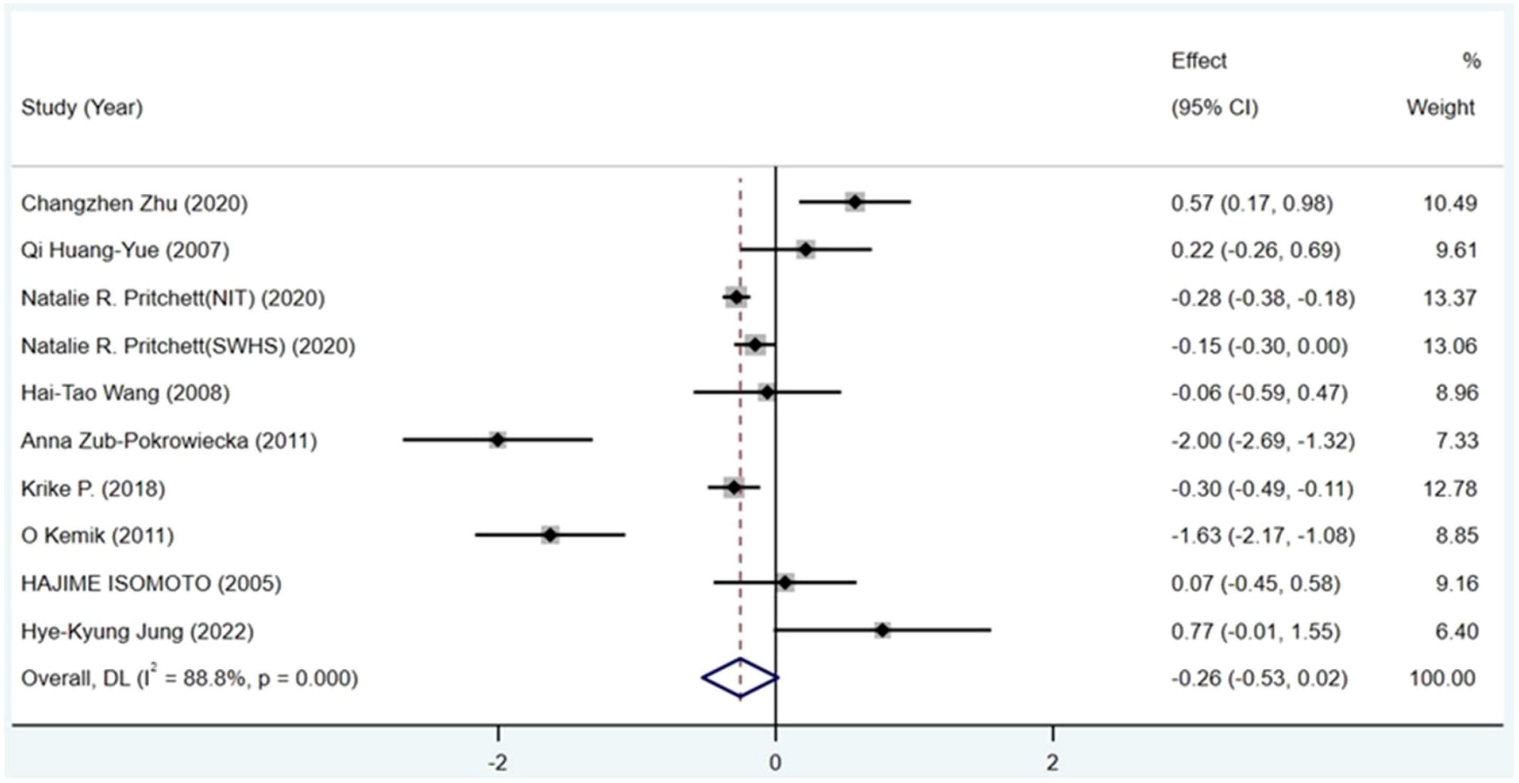
Forest plot showing the effect size of association between circulating ghrelin and gastric cancer. CI, Confidence interval. Summary estimates were analyzed using a random-effects model.

**Figure 3:**
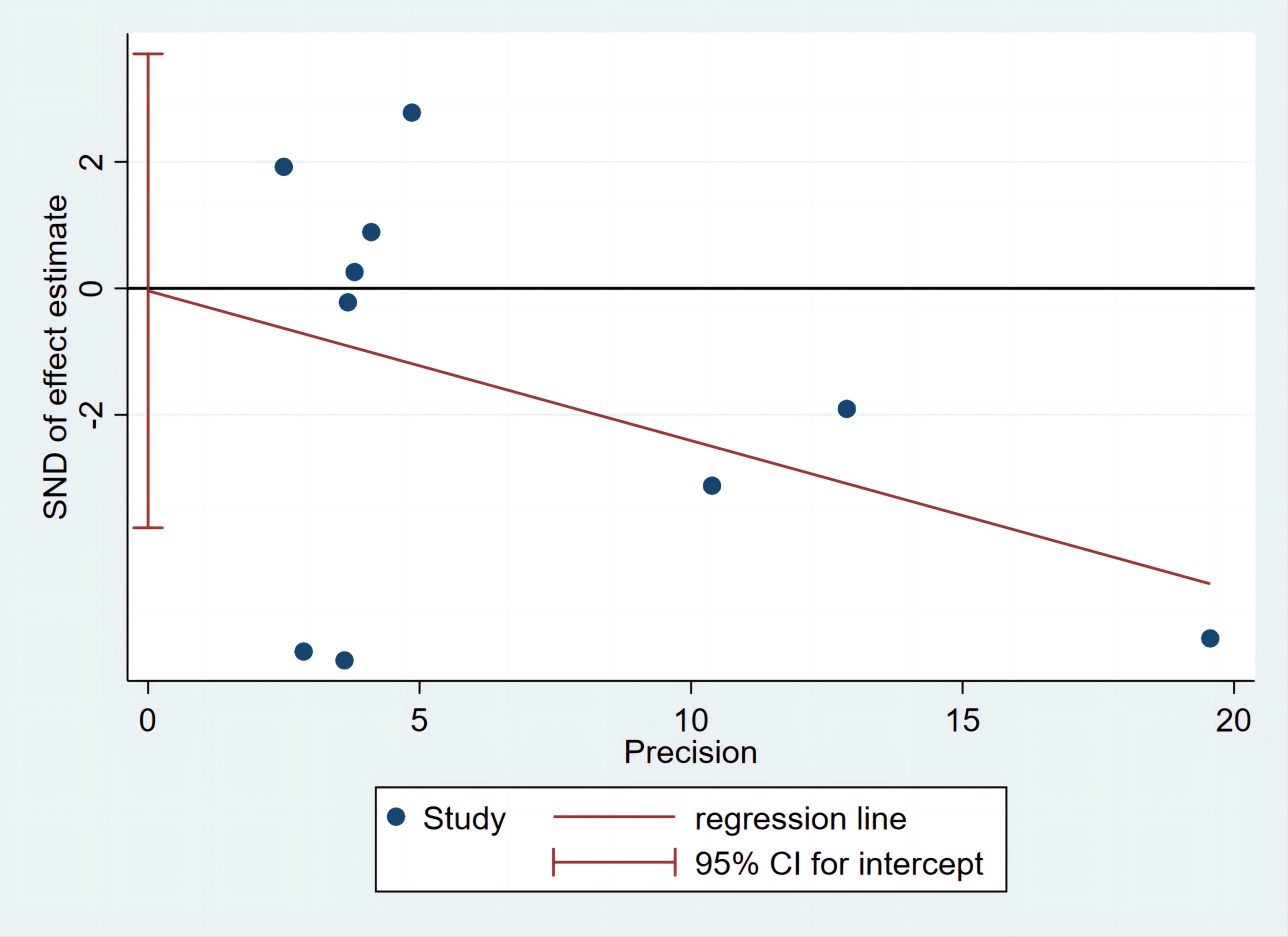
Egger’s publication bias plot.

**Figure 4:**
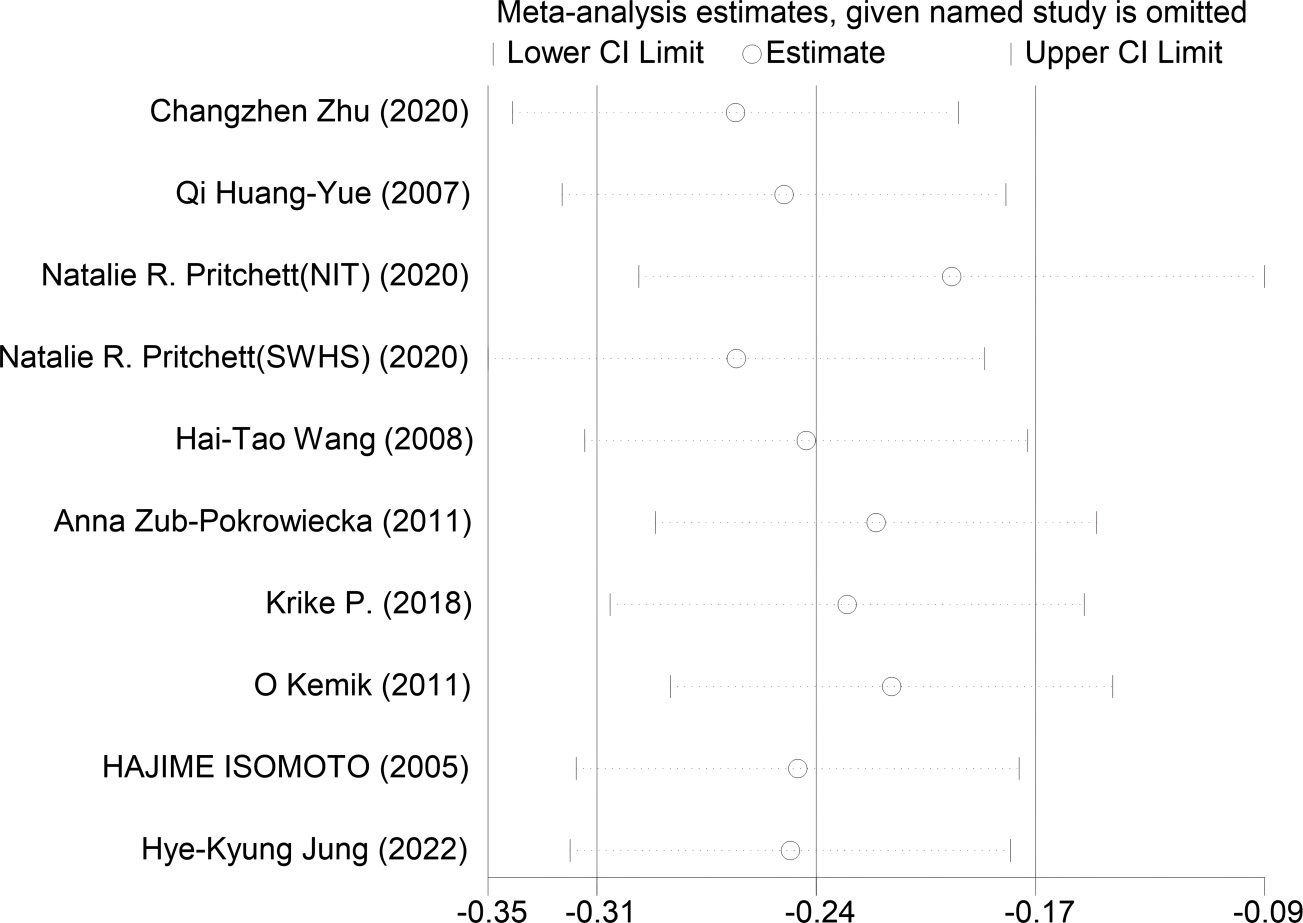
Impact analysis of a single study.

Ghrelin levels in patients were lower than in the control groups (**Figure 2**). (SMD=-0.255, 95%CI: -0.528 to 0.017). However, standard mean differences showed significant heterogeneity when analyzed using the random-effects model (I^2 ^= 88.8%, P < 0.00001). Publication bias was insignificant (**Figure 3**; Egger’s test: P= 0.981). Sensitivity analysis demonstrated the stability of our meta-analysis (**Figure 4**).

### Subgroup analysis

Subgroup analysis was performed to determine the factors affecting heterogeneity. Subgroup analyses classified by blood sample (serum or plasma) or race (Asian or European) showed no significant reductions or differences in heterogeneity (**Table 3**). This finding suggests that more information from different regions is needed for analysis.

**Table 3:** Subgroup analysis.

	Groups	Participants	Random effects (95% Cl)	I^2^ (%)
Overall	10	3,196	-0.255 (-0.528, 0.017)	88.8
**Blood sample**				
Plasma	9	3089	-0.349 (-0.618, -0.079)	87.6
Serum	1	107	0.572 (0.169, 0.975)	–
**Race**				
Asian	7	2633	0.062 (-0.175, 0.300)	77.7
European	3	563	-1.279 (-2.448, -0.110)	94.9

CI, Confidence interval.

### Discussion

Gastric cancer is a significant cause of cancer death worldwide, with a high mortality rate. Most gastric cancer is diagnosed at an advanced stage, with poor prognosis and limited treatment options (32). Gastric cancer outcomes are significantly related to the American Joint Committee on Cancer stage. The estimated adjusted 5-year survival rate after radical surgery was 41.3%, 82.9% for stage IA and stage IB, 62.8% for stage II, 17.8% for stage IIIA and stage IIIB, and 3.3% for stage IV (33). Therefore, early diagnosis is essential.

To our knowledge, the present study is the first meta-analysis to explore the association between circulating ghrelin levels and gastric cancer. Ghrelin levels were lower in gastric cancer patients than in controls. Our findings are consistent with those of Pritchett et al. (23). Ghrelin may be used as an early marker for gastric cancer screening after stratification of metabolic status including BMI and blood glucose of residents in high incidence areas of gastric cancer.

We performed subgroup analyses according to blood sample and race to examine sources of heterogeneity. There were no significant differences or reductions in heterogeneity. It should be noted that included studies were all from Europe or Asia. We attempted to obtain studies from other continents because of the limited number of studies but failed. Many other factors may influence circulating ghrelin levels.

(1) Ghrelin levels are inversely associated with BMI. Ghrelin levels are reduced in obese patients, suggesting a physiological adaptation to the positive energy balance associated with obesity (34, 35). Few studies in our meta-analysis adjusted for the association between circulating ghrelin levels and BMI, which varied among participants and influenced ghrelin levels. (2) Cancer-related cachexia causes weight loss (mainly from loss of skeletal muscle and body fat) and inflammation (36, 37). However, some studies in our meta-analysis did not separate gastric cancer patients due to cachexia (38, 39). (3) Gastric cancer patients follow different disease progressions, and few studies analyzed patients according to American Joint Committee on Cancer stage. (4) *H. pylori* is the main pathogen causing chronic active gastritis and plays a crucial role in gastric and duodenal ulcers and gastric cancer (40). *H. pylori* infection inhibits the expression of ghrelin active cells in the gastric mucosa, thereby reducing the level of ghrelin in the circulation. After eradication of *H. pylori* infection, ghrelin levels return to pre-infection levels (41–43). Similarly, few studies separated gastric cancer patients according to the presence or absence of *H. pylori* infection. (5) There are few related such studies, and ghrelin measurement methods are not standardized; furthermore, the normal range of ghrelin has not been determined. In many studies published in recent years, researchers have used methods to inhibit proteases followed by acidification of samples and obtained relatively accurate results (44, 45). Unfortunately, not all studies have used such preservation techniques. As in the table, experimental data measured by different experimenters differed significantly.

The surgical options for non-early operable gastric cancer are subtotal or total gastrectomy (46). Patients undergoing gastrectomy often suffer from weight loss, which reduces their quality of life, increasing the risk of contracting other diseases and affecting long-term survival. Several studies showed that ghrelin levels decreased significantly after gastrectomy but recovered over time. The concentrations of ghrelin decreased to 12–29% of the preoperative levels in total gastrectomy patients and 39–71% of the preoperative levels within three days of the gastrectomy (14).

Ghrelin is the only hormone known to promote appetite; researchers noted a possible role for ghrelin in cancer-associated cachexia (47). Two studies, one in patients after gastrectomy and one in patients with advanced cancer, demonstrated the beneficial effect of a large dose of exogenous ghrelin injection on increasing energy intake (43, 47). However, GHSR is known to express in many cancer cell types and may be upregulated in some cancers, including breast and colon cancer (48–50). Therefore, more studies are needed to demonstrate the safety of exogenous ghrelin injection.

Gastric cancer patients have different degrees of gastric atrophy, which is often accompanied by decreased ghrelin secretion (51). Therefore, the reduction of ghrelin may be a defense mechanism to limit the progression of gastric cancer. One limitation of this analysis is the small sample sizes of some studies, large-scale studies are needed to improve the accuracy of this meta-analysis. Many studies did not classify patients according to whether they had *H. pylori* infection or cachexia. Therefore, we could not make more accurate subgroup analyses based on *H. pylori* infection and cachexia. In addition, the literature search was performed using PubMed, Cochrane Library, EMBASE, and the Web of Science; Thus, language limitations may have increased the risk of publication bias.

In summary, we found that the circulating ghrelin levels were lower in patients with gastric cancer than in controls. Although the pooled results had high heterogeneity, the findings in trials with larger sample sizes agreed with our conclusion (23, 26). When ghrelin is used as an early marker for screening a large population in regions and countries with high incidence of gastric cancer, attention should be paid to factors affecting ghrelin levels, including the relationship between ghrelin and BMI and whether patients have *H. pylori* infection.

## Data availability statement

The original contributions presented in the study are included in the article/supplementary material. Further inquiries can be directed to the corresponding author.

## Author contributions

YuxW: Data curation, Formal analysis, Investigation, Methodology, Software, Writing- original draft, Writing- review & editing. CZ: Data curation, Investigation, Methodology, Software, Supervision, Writing- review & editing, Writing- original draft. QZ: Data curation, Investigation, Methodology, Software, Writing- review & editing. JY: Data curation, Methodology, Software, Writing- review & editing. YukW: Data curation, Software, Writing- review & editing. YX: Data curation, Supervision, Writing- review & editing. JD: Formal analysis, Funding acquisition, Methodology, Resources, Supervision, Writing- review & editing.

## References

[1] Sung H Ferlay J Siegel RL Laversanne M Soerjomataram I Jemal A . Global cancer statistics 2020: GLOBOCAN estimates of incidence and mortality worldwide for 36 cancers in 185 countries. CA: Cancer J Clin (2021) 71(3):209–49. doi: 10.3322/caac.21660 33538338

[2] McLean MH El-Omar EM . Genetics of gastric cancer. Nat Rev Gastroenterol hepatology. (2014) 11(11):664–74. doi: 10.1038/nrgastro.2014.143 25134511

[3] Smyth EC Nilsson M Grabsch HI van Grieken NC Lordick F . Gastric cancer. Lancet (London England) (2020) 396(10251):635–48. doi: 10.1016/S0140-6736(20)31288-5 32861308

[4] Van Cutsem E Sagaert X Topal B Haustermans K Prenen H . Gastric cancer. Lancet (London England) (2016) 388(10060):2654–64. doi: 10.1016/S0140-6736(16)30354-3 27156933

[5] Machlowska J Baj J Sitarz M Maciejewski R Sitarz R . Gastric cancer: epidemiology, risk factors, classification, genomic characteristics and treatment strategies. Int J Mol Sci (2020) 21(11):4012. doi: 10.3390/ijms21114012 PMC7312039 32512697

[6] Lamers CB Jansen JB Woutersen RA . Cholecystokinin and gastrointestinal cancer. J Steroid Biochem Mol Biol (1990) 37(6):1069–72. doi: 10.1016/0960-0760(90)90467-y 2285582

[7] Morris DL Watson SA Durrant LG Harrison JD . Hormonal control of gastric and colorectal cancer in man. Gut (1989) 30(4):425–9. doi: 10.1136/gut.30.4.425 PMC1434051 2653970

[8] Chopin L Walpole C Seim I Cunningham P Murray R Whiteside E . Ghrelin and cancer. Mol Cell Endocrinol. (2011) 340(1):65–9. doi: 10.1016/j.mce.2011.04.013 21616120

[9] Kojima M Hosoda H Date Y Nakazato M Matsuo H Kangawa K . Ghrelin is a growth-hormone-releasing acylated peptide from stomach. Nature (1999) 402(6762):656–60. doi: 10.1038/45230 10604470

[10] Kojima M Kangawa K . Ghrelin: structure and function. Physiol Rev (2005) 85(2):495–522. doi: 10.1152/physrev.00012.2004 15788704

[11] Date Y Kojima M Hosoda H Sawaguchi A Mondal MS Suganuma T . Ghrelin, a novel growth hormone-releasing acylated peptide, is synthesized in a distinct endocrine cell type in the gastrointestinal tracts of rats and humans. Endocrinology (2000) 141(11):4255–61. doi: 10.1210/endo.141.11.7757 11089560

[12] van der Lely AJ Tschöp M Heiman ML Ghigo E . Biological, physiological, pathophysiological, and pharmacological aspects of ghrelin. Endocrine Rev (2004) 25(3):426–57. doi: 10.1210/er.2002-0029 15180951

[13] Pacifico L Poggiogalle E Costantino F Anania C Ferraro F Chiarelli F . Acylated and nonacylated ghrelin levels and their associations with insulin resistance in obese and normal weight children with metabolic syndrome. Eur J Endocrinol. (2009) 161(6):861–70. doi: 10.1530/EJE-09-0375 19773372

[14] Takiguchi S Takata A Murakami K Miyazaki Y Yanagimoto Y Kurokawa Y . Clinical application of ghrelin administration for gastric cancer patients undergoing gastrectomy. Gastric Cancer Off J Int Gastric Cancer Assoc Japanese Gastric Cancer Assoc (2014) 17(2):200–5. doi: 10.1007/s10120-013-0300-8 24253567

[15] Shintani M Ogawa Y Ebihara K Aizawa-Abe M Miyanaga F Takaya K . Ghrelin, an endogenous growth hormone secretagogue, is a novel orexigenic peptide that antagonizes leptin action through the activation of hypothalamic neuropeptide Y/Y1 receptor pathway. Diabetes (2001) 50(2):227–32. doi: 10.2337/diabetes.50.2.227 11272130

[16] Li WG Gavrila D Liu X Wang L Gunnlaugsson S Stoll LL . Ghrelin inhibits proinflammatory responses and nuclear factor-kappaB activation in human endothelial cells. Circulation (2004) 109(18):2221–6. doi: 10.1161/01.CIR.0000127956.43874.F2 15117840

[17] Kotta AS Kelling AS Corleto KA Sun Y Giles ED . Ghrelin and cancer: examining the roles of the ghrelin axis in tumor growth and progression. Biomolecules (2022) 12(4):483. doi: 10.3390/biom12040483 PMC9032665 35454071

[18] Soleyman-Jahi S Sadeghi F Pastaki Khoshbin A Khani L Roosta V Zendehdel K . Attribution of ghrelin to cancer; attempts to unravel an apparent controversy. Front Oncol (2019) 9:1014. doi: 10.3389/fonc.2019.01014 31681567 PMC6805778

[19] Au CC Furness JB Brown KA . Ghrelin and breast cancer: emerging roles in obesity, estrogen regulation, and cancer. Front Oncol (2016) 6:265. doi: 10.3389/fonc.2016.00265 28119851 PMC5220482

[20] Ginter G Ceranowicz P Warzecha Z . Protective and healing effects of ghrelin and risk of cancer in the digestive system. Int J Mol Sci (2021) 22(19):10571. doi: 10.3390/ijms221910571 PMC8509076 34638910

[21] Zub-Pokrowiecka A Rembiasz K Konturek PC Budzyński A Konturek SJ Winiarski M . Ghrelin and gastrin in advanced gastric cancer before and after gastrectomy. World J Gastroenterol. (2011) 17(4):449–58. doi: 10.3748/wjg.v17.i4.449 PMC3027011 21274374

[22] Wang HT Lu QC Wang Q Wang RC Zhang Y Chen HL . Role of the duodenum in regulation of plasma ghrelin levels and body mass index after subtotal gastrectomy. World J Gastroenterol. (2008) 14(15):2425–9. doi: 10.3748/wjg.14.2425 PMC2705102 18416474

[23] Pritchett NR Maziarz M Shu XO Kamangar F Dawsey SM Fan JH . Serum ghrelin and esophageal and gastric cancer in two cohorts in China. Int J Cancer (2020) 146(10):2728–35. doi: 10.1002/ijc.32597 PMC7477842 31351006

[24] Zhu C Liu Y Kang W Zhang Z Zeng Z Liu D . Exploration of the role of serum ghrelin in the diagnosis and treatment of digestive tract Malignancies. J Int Med Res (2020) 48(5):300060520920441. doi: 10.1177/0300060520920441 32366148 PMC7221476

[25] Isomoto H Ueno H Saenko VA Mondal MS Nishi Y Kawano N . Impact of Helicobacter pylori infection on gastric and plasma ghrelin dynamics in humans. Am J Gastroenterol. (2005) 100(8):1711–20. doi: 10.1111/j.1572-0241.2005.41492.x 16086706

[26] Krike P Rudzite D Polaka I Kojalo I Isajevs S Leja M . Decrease of trefoil factor 3 and ghrelin in gastric cancer patients. Helicobacter (2018) 23:e12525. doi: 10.1111/hel.12525

[27] Bowden J Tierney JF Copas AJ Burdett S . Quantifying, displaying and accounting for heterogeneity in the meta-analysis of RCTs using standard and generalised Q statistics. BMC Med Res Methodol. (2011) 11:41. doi: 10.1186/1471-2288-11-41 21473747 PMC3102034

[28] Kemik O Kemik AS Begenik H Erdur FM Emre H Sumer A . The relationship among acute-phase responce proteins, cytokines, and hormones in various gastrointestinal cancer types patients with cachectic. Hum Exp Toxicol. (2012) 31(2):117–25. doi: 10.1177/0960327111417271 21803781

[29] Isomoto H Ueno H Nishi Y Yasutake T Tanaka K Kawano N . Circulating ghrelin levels in patients with various upper gastrointestinal diseases. Digestive Dis Sci (2005) 50(5):833–8. doi: 10.1007/s10620-005-2648-z 15906753

[30] Huang Q Fan YZ Ge BJ Zhu Q Tu ZY . Circulating ghrelin in patients with gastric or colorectal cancer. Digestive Dis Sci (2007) 52(3):803–9. doi: 10.1007/s10620-006-9508-3 17245626

[31] Jung HK Tae CH Lee HA Lee KE Moon CM Kim SE . Association between gut regulatory hormones and post-operative weight loss following gastrectomy in patients with gastric cancer. J Neurogastroenterol Motil. (2022) 28(3):409–17. doi: 10.5056/jnm21145 PMC9274481 35799234

[32] Necula L Matei L Dragu D Neagu AI Mambet C Nedeianu S . Recent advances in gastric cancer early diagnosis. World J Gastroenterol. (2019) 25(17):2029–44. doi: 10.3748/wjg.v25.i17.2029 PMC6506585 31114131

[33] Spataro V Genoni M Maurer C Müller W . [Stomach cancer: 10 years experiences with surgical treatment and possibilities for improving the prognosis]. Helv Chirurgica Acta (1993) 59(4):589–95. 8473176

[34] Park HS Lee KU Kim YS Park CY . Relationships between fasting plasma ghrelin levels and metabolic parameters in children and adolescents. Metabolism: Clin experimental. (2005) 54(7):925–9. doi: 10.1016/j.metabol.2005.02.007 15988702

[35] Tschöp M Weyer C Tataranni PA Devanarayan V Ravussin E Heiman ML . Circulating ghrelin levels are decreased in human obesity. Diabetes (2001) 50(4):707–9. doi: 10.2337/diabetes.50.4.707 11289032

[36] Baracos VE Martin L Korc M Guttridge DC Fearon KCH . Cancer-associated cachexia. Nat Rev Dis Primers (2018) 4:17105. doi: 10.1038/nrdp.2017.105 29345251

[37] Argilés JM Busquets S Stemmler B López-Soriano FJ . Cancer cachexia: understanding the molecular basis. Nat Rev Cancer (2014) 14(11):754–62. doi: 10.1038/nrc3829 25291291

[38] Akamizu T Kangawa K . Ghrelin for cachexia. J Cachexia Sarcopenia Muscle (2010) 1(2):169–76. doi: 10.1007/s13539-010-0011-5 PMC3060649 21475698

[39] Garcia JM Garcia-Touza M Hijazi RA Taffet G Epner D Mann D . Active ghrelin levels and active to total ghrelin ratio in cancer-induced cachexia. J Clin Endocrinol Metab (2005) 90(5):2920–6. doi: 10.1210/jc.2004-1788 15713718

[40] Marshall BJ McGechie DB Francis GJ Utley PJ . Pyloric campylobacter serology. Lancet (London England) (1984) 2(8397):281. doi: 10.1016/S0140-6736(84)90318-0 6146825

[41] Tatsuguchi A Miyake K Gudis K Futagami S Tsukui T Wada K . Effect of Helicobacter pylori infection on ghrelin expression in human gastric mucosa. Am J Gastroenterol (2004) 99(11):2121–7. doi: 10.1111/j.1572-0241.2004.30291.x 15554990

[42] Isomoto H Ueno H Nishi Y Wen CY Nakazato M Kohno S . Impact of Helicobacter pylori infection on ghrelin and various neuroendocrine hormones in plasma. World J Gastroenterol (2005) 11(11):1644–8. doi: 10.3748/wjg.v11.i11.1644 PMC4305946 15786542

[43] Adachi S Takiguchi S Okada K Yamamoto K Yamasaki M Miyata H . Effects of ghrelin administration after total gastrectomy: a prospective, randomized, placebo-controlled phase II study. Gastroenterology (2010) 138(4):1312–20. doi: 10.1053/j.gastro.2009.12.058 20060830

[44] Lund LH Hage C Pironti G Thorvaldsen T Ljung-Faxén U Zabarovskaja S . Acyl ghrelin improves cardiac function in heart failure and increases fractional shortening in cardiomyocytes without calcium mobilization. Eur Heart J (2023) 44(22):2009–25. doi: 10.1093/eurheartj/ehad100 PMC10256198 36916707

[45] Kleftaki SA Simati S Amerikanou C Gioxari A Tzavara C Zervakis GI . Pleurotus eryngii improves postprandial glycaemia, hunger and fullness perception, and enhances ghrelin suppression in people with metabolically unhealthy obesity. Pharmacol Res (2022) 175:105979. doi: 10.1016/j.phrs.2021.105979 34798266

[46] Joshi SS Badgwell BD . Current treatment and recent progress in gastric cancer. CA: Cancer J Clin (2021) 71(3):264–79. doi: 10.3322/caac.21657 PMC9927927 33592120

[47] Blum D de Wolf-Linder S Oberholzer R Brändle M Hundsberger T Strasser F . Natural ghrelin in advanced cancer patients with cachexia, a case series. J Cachexia Sarcopenia Muscle (2021) 12(2):506–16. doi: 10.1002/jcsm.12659 PMC8061403 33452750

[48] Chopin LK Seim I Walpole CM Herington AC . The ghrelin axis–does it have an appetite for cancer progression? Endocrine Rev (2012) 33(6):849–91. doi: 10.1210/er.2011-1007 22826465

[49] Waseem T Javaid Ur R Ahmad F Azam M Qureshi MA . Role of ghrelin axis in colorectal cancer: a novel association. Peptides. (2008) 29(8):1369–76. doi: 10.1016/j.peptides.2008.03.020 18471933

[50] Grönberg M Nilsson C Markholm I Hedenfalk I Blomqvist C Holmberg L . Ghrelin expression is associated with a favorable outcome in male breast cancer. Sci Rep (2018) 8(1):13586. doi: 10.1038/s41598-018-31783-x 30206250 PMC6134078

[51] Uemura N Okamoto S Yamamoto S Matsumura N Yamaguchi S Yamakido M . Helicobacter pylori infection and the development of gastric cancer. New Engl J Med (2001) 345(11):784–9. doi: 10.1056/NEJMoa001999 11556297

